# Somatic comorbidity in anorexia nervosa: First results of a 21-year follow-up study on female inpatients

**DOI:** 10.1186/1751-0759-6-4

**Published:** 2012-02-02

**Authors:** Laurence Erdur, Bettina Kallenbach-Dermutz, Vicky Lehmann, Frank Zimmermann-Viehoff, Werner Köpp, Cora Weber, Hans-Christian Deter

**Affiliations:** 1 Department of Psychosomatic Medicine and Psychotherapy, Charité Universitätsmedizin, Campus Benjamin Franklin, Berlin, Germany; 2 Department of Medical Psychology and Neuropsychology, Centre of Research on Psychology in Somatic diseases (CoRPS), Tilburg University, Tilburg, The Netherlands

**Keywords:** anorexia nervosa, long-term course, somatic comorbidity, mortality

## Abstract

**Background:**

Anorexia nervosa is a severe psychosomatic disease with somatic complications in the long-term course and a high mortality rate. Somatic comorbidities independent of anorexia nervosa have rarely been studied, but pose a challenge to clinical practitioners. We investigated somatic comorbidities in an inpatient cohort and compared somatically ill anorexic patients and patients without a somatic comorbidity. In order to evaluate the impact of somatic comorbidity for the long-term course of anorexia nervosa, we monitored survival in a long-term follow-up.

**Method:**

One hundred and sixty-nine female inpatients with anorexia nervosa were treated at the Charité University Medical Centre, Campus Benjamin Franklin, Berlin, between 1979 and 2011. We conducted retrospective analyses using patient's medical and psychological records. Information on survival and mortality were required through the local registration office and was available for one hundred patients. The mean follow-up interval for this subgroup was m = 20.9 years (sd = 4.7, min = 13.3, max = 31.6, range = 18.3). We conducted survival analysis using cox regression and included somatic comorbidity in a multivariate model.

**Results:**

N = 41 patients (24.3%) showed a somatic comorbidity, n = 13 patients (7.7%) showed somatic comorbidities related to anorexia nervosa and n = 26 patients (15.4%) showed somatic comorbidities independent of anorexia nervosa, n = 2 patients showed somatic complications related to other psychiatric disorders. Patients with a somatic comorbidity were significantly older (m = 29.5, sd = 10.3 vs m = 25.0, sd = 8.7; p = .006), showed a later anorexia nervosa onset (m = 24.8, sd = 9.9 vs. m = 18.6, sd = 5.1; p < .000) and a longer duration of treatment in our clinic (m = 66.6, sd = 50.3 vs. m = 50.0, sd = 47; p = .05) than inpatients without somatic comorbidity. Out of 100 patients, 9 patients (9%) had died, on average at age of m = 37 years (sd = 9.5). Mortality was more common among inpatients with somatic comorbidity (n = 6, 66.7%) than among inpatients without a somatic disease (n = 3, 33.3%; p = .03). Somatic comorbidity was a significant coefficient in a multivariate survival model (B = 2.32, p = .04).

**Conclusion:**

Somatic comorbidity seems to be an important factor for anorexia nervosa outcome and should be included in multivariate analyses on the long-term course of anorexia nervosa as an independent variable. Further investigations are needed in order to understand in which way anorexia nervosa and a somatic disease can interact.

## Background

Anorexia nervosa is a severe psychosomatic disease with a lifetime prevalence of 1.2%-2.2% [1]. The Standardized Mortality Ratio (SRM) for anorexia nervosa is 5.9, as recently published meta-analyses showed [2]. Thus, anorexia nervosa is the psychosomatic disease with the highest rate of mortality. Severe somatic complications related to poor outcome of anorexia nervosa (eg. osteoporosis, renal insufficiency) have been observed in the long-term course [3,4]. These somatic complications result from the anorexia inherent symptoms, such as permanent underweight, amenorrhoea or purging behaviour. However, little is known about the course of illness in patients who suffer from a somatic disease independent from anorexia nervosa, such as chronic internal diseases like diabetes mellitus or inflammatory bowel diseases. This subgroup of anorexic patients is mostly excluded in empirical follow-up studies in order to create homogeneous samples. Apart from case reports [5-7], we found only one empirical study in which this specific subgroup of patients was mentioned [8] which reported a very poor long-term prognosis and a high mortality rate. In clinical practice, these patients require intensive and integrative care where interacting effects of the somatic and psychosomatic disease constitute a major challenge for therapeutic interventions.

Summing up the existing case reports on anorexia nervosa patients with a comorbid somatic disease, the following possible mechanisms of interaction can be derived:

■ Anorexia nervosa patients often show low compliance and denial of illness [9]. This might also complicate the treatment of a somatic disease (e. g. Asthma, [10])

■ Anorexia nervosa symptoms, such as permanent underweight, influence the immune system and lead to frequent infections (e. g. Tuberculosis; [11])

■ The psychological (mal-) adaptation to the onset of a somatic disease can trigger the development of anorexia nervosa (e. g. Diabetes mellitus type one; [12])

■ A somatic disease related to weight changes or alterations in food intake can "hide" a comorbid eating disorder (e. g. Crohn's disease; [7])

■ Somatic complications resulting from anorexia nervosa aggravate the course of a somatic disease (e. g. Lupus erythematodes; [6])

Until now, it is not clear which role somatic comorbidity plays in the long-term course of anorexia nervosa, since it is not known which somatic diseases most frequently co-occur with anorexia nervosa and if there are specific features for these patients. Therefore, we investigated basic clinical characteristics and somatic comorbidity of anorexic inpatients during psychosomatic therapy and monitored survival and mortality in a long-term follow-up.

## Method

### Sample

One hundred and sixty-nine consecutive female inpatients with a consensus diagnosis of anorexia nervosa according to DSM-IV criteria were treated between 1979 and 2010 at the psychosomatic department of the Charité University Medical Centre, Campus Benjamin Franklin, Berlin. The mean age was m = 6.2 years (sd = 9.3), the mean body mass index (BMI) at admission was m = 15.2 (sd = 2.1). N = 90 patients (53.3%) showed the purging and n = 74 (43.8%) the restrictive anorexia nervosa subtype. The mean duration of illness at first presentation in our clinic was m = 6.3 years (sd = 7.6).

### Treatment

All patients followed the same psychosomatic treatment, focusing on acute psychological and somatic stabilization: Beside internal medical diagnostic and therapeutic measurements for stabilization of weight and eating behaviour, and p.e. normalization of electrolytes, we applied individual and group therapy, art therapy, concentrative movement therapy, and relaxation. On average, the treatment took m = 54.2 days (sd = 48.2).

### Instruments

We conducted retrospective analyses using differentiated medical and psychological records of the patients from the beginning of inpatient treatment (T_0_). Medical data included somatic comorbidities, medication, blood test results, and standardized measurements of weight and height. Psychological records provided data on duration and onset of illness, socio-demographic data and psychiatric comorbidity.

### Assessment of mortality

Data on mortality were required through the appropriate registration office between 2010 and 2011 (T_1_). At time of publication, data on N = 100 patients was available. The mean follow-up interval for this sample was m = 20.9 years (sd = 4.8).

### Statistical analysis

We conducted parametric tests such as the Students T-Tests for independent samples or the Chi^2^-Test for nominal scaled data. For mortality, we conducted survival analysis using cox regression and included somatic comorbidity as an independent variable in a multivariate model using stepwise inclusion of parameters (inclusion criterion p < .05). Calculations were done with SPSS 19.0. Coefficients are considered significant if the respective p-values are less than α = 0.05.

### Ethical approval

The study was approved by the local ethical committee of the Charité University Medical Centre, Campus Benjamin Franklin, Berlin, Germany (application number EA4/073/08).

## Results

### Somatic comorbidity

Somatic comorbidity at T_0 _was present in N = 41 patients (24.3%). N = 13 patients (7.7%) showed somatic comorbidities related to anorexia nervosa, predominantly internal diseases (n = 9). N = 26 patients (15.4%), showed somatic comorbidities independent of anorexia nervosa, also most frequently in the field of internal medicine (n = 9). N = 2 patients showed somatic complications related to other psychiatric disorders (one case with abscesses due to an artificial disorder and one case with hepatitis resulting from chronic alcohol abuse). Somatic comorbidities are presented in table 1.

**Table 1:** Somatic comorbidities in N = 169 female inpatients with anorexia nervosa

Somatic comorbidities related to anorexia nervosa			
	**n**	**%**	

Internal Medicine	9	5.3	Nephropathie (1) Renal insufficiency (2) Sinus Tachycardia (1) Mitral valve prolapse (1) Endocarditis (1) Alimentary gastritis (1) Ileus (1), Duodenitis (1)

Dentistry	1	0.6	Multiple Dental Destructions (1)

Orthopaedy	3	1.8	Osteoporosis (3)

Total	13	7.7	

**Somatic comorbidities independent of anorexia nervosa**			

	n	%	

Internal Medicine	13	7.7	Asthma (3) COPD (1) Diabetes mellitus Type 1 (2) EBV Infection (1) Ulcera ventriculi (1) Inflammatory Bowel Diseases (3) Thalassemia (1) Nephrolithiasis (1)

Dermatology	7	4.1	Atopy (2) Eczema (1) Urticaria (1) Tinea pedis (1) Keratosis (1) Psoriasis (1)

Gynaecology	1	0.6	Polycystic Ovary Syndrome (1)

Haematology	2	1.2	Breast Cancer (1) Anal Cancer (1)

Neurology	1	0.6	Epilepsy (1)

Othopaedy	3	1.8	Comminuted Fracture (1) Scoliosis (1) Hip Dysplasia (1)

Total	26	15.4	

**Somatic comorbidities related to (other) psychiatric disorders**			

	n	%	

Surgery	1	0.6	Abscesses (Artificial Disorder)

Internal Medicine	1	0.6	Hepatitis (toxic, related to alcohol abuse)

Total	2	1.2	

**Total**	**41**	**24.3**	

### Specific features of anorexia nervosa inpatients with and without somatic comorbidity

At T_0_, anorexia nervosa inpatients with somatic comorbidity were significantly older (m = 29.5, sd = 10.3) than inpatients without a somatic comorbidity (m = 25.0, sd = 8.7; p = .006), showed a later anorexia nervosa onset (m = 24.8, sd = 9.9 vs. m = 18.6, sd = 5.1; p < .000) and a longer duration of treatment in our clinic (m = 66.6, sd = 50.3 vs. m = 50.0, sd = 47; p = .05). No differences were observed with regard to BMI at admission, BMI change during treatment or duration of anorexia nervosa before presentation in our clinic. Education status did not differ between groups, nor did psychiatric comorbidity. No differences were observed for frequencies of anorexia nervosa subtype. See table 2 for detailed statistics.

**Table 2:** Specific features of anorexia nervosa inpatients with and without somatic comorbidity

		m	sd	p
Age at admission (T_0_)	AN* with somatic comorbidity	29.5	10.3	.006**

	AN without somatic comorbidity	25.0	8.7	

Duration of treatment (days)	AN with somatic comorbidity	66.6	50.3	.05*

	AN without somatic comorbidity	50.0	47.0	

BMI at admission (T_0_)	AN with somatic comorbidity	14.8	1.8	ns

	AN without somatic comorbidity	15.3	2.1	

BMI change during treatment	AN with somatic comorbidity	0.7	1.2	ns

	AN without somatic comorbidity	0.6	0.9	

Age at onset of AN	AN with somatic comorbidity	24.8	9.9	< .000***

	AN without somatic comorbidity	18.6	5.1	

Duration of AN (T_0_)	AN with somatic comorbidity	5.1	6.6	ns

	AN without somatic comorbidity	6.7	7.8	

Subtype of AN				

restrictive	AN with somatic comorbidity	17	41.5%	ns

	AN without somatic comorbidity	57	46.3%	

purging	AN with somatic comorbidity	24	58.5%	

	AN without somatic comorbidity	66	53.7%	

Psychiatric comorbidity				

Depressive disorders	AN with somatic comorbidity	11	39.3%	ns

	AN without somatic comorbidity	28	45.2%	

Anxiety disorders	AN with somatic comorbidity	6	21.4%	ns

	AN without somatic comorbidity	12	19.4%	

OCD**	AN with somatic comorbidity	4	14.3%	ns

	AN without somatic comorbidity	5	7.9%	

Personality Disorders	AN with somatic comorbidity	3	27.3%	ns

	AN without somatic comorbidity	7	43.8%	

Substance Abuse	AN with somatic comorbidity	5	17.2%	ns

	AN without somatic comorbidity	10	16.1%	

Total	AN with somatic comorbidity	16	38.1%	ns

	AN without somatic comorbidity	33	26.0%	

*AN: Anorexia nervosa

**OCD: Obsessive Compulsive Disorder

### Mortality

**Figure 1:**
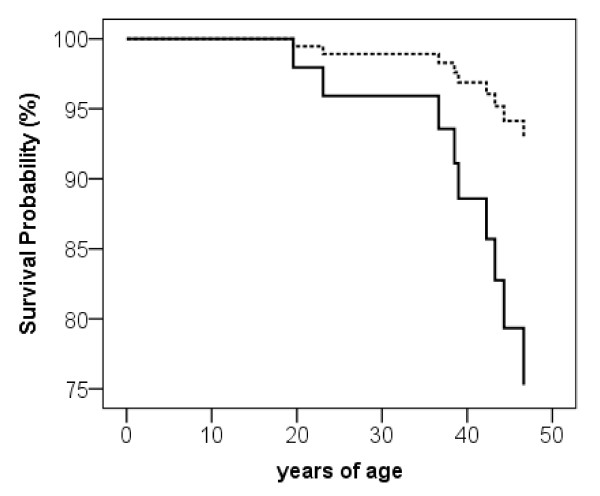
**Survival in 100 female inpatients with anorexia nervosa**. somatic comorbidity. no somatic comorbidity.

At time of publication, data on 100 patients treated between 1979 and 1997 was available. Out of these 100 patients, 9 patients (9%) had died. Age of death was m = 37.0 years (sd = 9.5, range 19.6 - 46.6 years), and the interval between end of treatment in our department and death was m = 7.9 years (sd = 7.2). One patient died shortly after discharge (the patient refused to continue the psychosomatic treatment and left our clinic against medical advice; see Köpp et al. for a detailed case report [13]). Mortality was significantly more common among inpatients with somatic comorbidity (n = 6, 66.7%) than among inpatients without a somatic disease (n = 3, 33.3%; p = .03). Mortality was also associated with higher frequencies of substance abuse (n = 4, 44.4% vs. n = 11, 13.3%; p = .02; laxative use is not included). No differences with regard to age at T_0_, onset of illness, purging behaviour, BMI at admission or weight gain during treatment were observed. See table 3 for detailed statistics and Figure 1 for results of the survival analyses.

**Table 3:** Specific features of N = 100 anorexia nervosa inpatients in a 21-year follow-up (N = 91 alive and N = 9 deceased)

		m	sd	p
Age at admission (T_0_)	Alive	23.5	5.7	ns

	Deceased	29.0	9.0	

Duration of treatment (days)	Alive	75.7	40.7	ns

	Deceased	60.3	50.1	

BMI at admission (T_0_)	Alive	14.9	1.9	ns

	Deceased	15.8	2.4	

BMI change during treatment	Alive	0.8	1.1	ns

	Deceased	0.3	1.7	

Age at onset of anorexia nervosa	Alive	19.4	4.6	ns

	Deceased	26.0	10.1	

Duration of anorexia nervosa (T_0_)	Alive	3.8	4.2	ns

	Deceased	3.8	2.7	

Purging Behaviour		N	%	

	Alive	58	67.4%	ns

	Deceased	7	87.5%	

Psychiatric comorbidity	Alive	43	47.8%	ns

	Deceased	6	66.7%	

Substance abuse	Alive	11	13.3%	.02*

	Deceased	4	44.4%	

Somatic comorbidity	Alive	25	27.8%	.03*

	Deceased	6	66.7%	


In a survival analysis using cox regression, somatic comorbidity proved to be significant (B = 2.32, p = .04) and was included as the only significant coefficient variable in a significance model (Chi^2 ^= 6.6, p = .02) using stepwise inclusion of eleven parameters (excluded: age at T_0_, duration of treatment, BMI at T_0_, BMI gain during treatment, onset of anorexia nervosa, duration of anorexia nervosa at T_0_, psychiatric comorbidity, substance abuse, type of anorexia nervosa restrictive/purging).

## Discussion

In our clinical study on 169 female anorexia nervosa inpatients, we found 24.3% of the patients with a comorbid somatic disease. These diseases were partly related (7.7%) and more frequently unrelated (15.4%) to anorexia nervosa. For both subgroups, internal diseases were observed most frequently. Overall, diverse somatic comorbidities from different medical fields were reported. Somatically ill anorexia nervosa patients showed higher age at the time of treatment, a later onset of anorexia nervosa and had a longer duration of treatment. Mortality was observed in nine of one hundred patients and was significantly higher in somatically ill patients. The relationship between mortality and somatic comorbidity was also statistically shown in a survival analysis, revealing somatic comorbidity as a significant coefficient.

The small sample of deceased anorexia nervosa inpatients did not allow us to conduct more sophisticated statistical analyses, such as regression analyses or survival analyses. The interpretation and generalization of our results is limited. No information on the causes of death was available for us. Therefore, we cannot draw confident causal conclusions between the observed correlation of mortality and somatic comorbidity.

The relatively high rate of somatically ill anorexia nervosa inpatients can be explained with the specific referral procedure to our university medical centre. Moreover, the intensive cooperation between our psychosomatic department and other units, such as the gastroenterological, neurological, and endocrinological departments, probably led to frequent referrals of patients with somatic illnesses.

Long-term mortality in anorexia nervosa has been intensively studied, and mortality rates vary together with the follow-up interval [2]. Data on the outcome of anorexia nervosa after more than 15 years is rare, and our follow-up interval of 21 years can be considered as relatively long. The observed mortality rate in our sample is in the rage of mortality rates to be expected with regard to the existing literature. However, we cannot confirm the reported correlations of laxative use/purging behaviour, BMI, duration of illness/onset, and psychiatric comorbidity. We only can confirm the relationship between substance abuse and mortality, which has also often been described in former studies [2]. The reason for this discrepancy is probably our small and specific sample.

To our knowledge, the present study is the first empirical sample that describes anorexia nervosa inpatients with a somatic comorbidity. The correlation with mortality might at least indicate a poor outcome for inpatients in this subsample, which confirms the clinical impression of medical practitioners treating anorexia nervosa patients. The relatively high co-occurrence of anorexia nervosa and somatic diseases in all medical fields is probably an issue that medical professionals are not overly aware of in clinical practice. Especially, in cases of patients where the somatic disease is associated with alterations in food intake and weight, such as inflammatory bowel diseases, medical professionals may not consider the possible existence of a comorbid anorexia nervosa. Moreover, we observed a high age and a late onset of anorexia nervosa in this subgroup, features that may not "fit" into the clinical idea medical practitioners have of the "typical" anorexic individual. The high rate of mortality in this group could reflect the "result" of this supposed problematic constellation.

To assure a good therapeutic outcome for anorexia nervosa patients with a somatic comorbidity, a high level of interdisciplinary treatment including all medical fields and early interventions by professionals in the field of psychosomatic medicine is needed. This group of patients might be at a specific risk for particularly poor long-term outcome. Further studies are needed to confirm these findings.

## Conclusion

Anorexia nervosa patients with a somatic comorbidity pose a challenge to medical professionals and might be at risk for poor long-term outcome. Further long-term studies on anorexia nervosa should include somatic comorbidity as an independent variable in a multivariate design.

## Competing interests

This study was founded by grants of the Christina-Barz-Stiftung (Stifterverband der deutschen Wissenschaft).

## Authors' contributions

LE collected the data of former inpatients, required data on mortality and wrote the main parts of this manuscript; BKD was involved in the treatment of the described inpatients; VL carried out statistical analyses; WK was involved in drafting the manuscript; FZV and CW contributed to the interpretation of data; HCD designed the study. All authors approved to the final version of the manuscript.
